# Low-frequency ultrasonic treatment: A potential strategy to improve the flavor of fresh watermelon juice

**DOI:** 10.1016/j.ultsonch.2022.106238

**Published:** 2022-11-23

**Authors:** Fan Yang, Chunhe Shi, Lichang Yan, Ying Xu, Yixin Dai, Shuang Bi, Ye Liu

**Affiliations:** Beijing Engineering and Technology Research Center of Food Additives, Beijing Advanced Innovation Center for Food Nutrition and Human Health, School of Food and Health, Beijing Technology and Business University (BTBU), Beijing 100048, China

**Keywords:** SPME, solid-phase microextraction, SAFE, solvent-assisted flavor evaporation, GC-O-MS, gas chromatography-olfactometry-mass spectrometry, RI, retention index, AEDA, aroma extraction dilution analysis, OAV, odor activity value, GC×GC-O-MS, comprehensive two-dimensional gas chromatography-olfactometry-mass spectrometry, FW, fresh watermelon juice, UW, ultrasonic treated watermelon juice, DVB/CAR/PDMS, divinylbenzene/carboxen/polydimethylsiloxane, RT, retention time, Watermelon juice, Ultrasonic treatment, Molecular sensory science, Flavor

## Abstract

A molecular sensory science approach was used to explore the effects of ultrasonic treatment on aroma compounds of watermelon juice. Watermelon juice was submitted to ultrasonic power at 325 W for 20 min. Ultrasonic treatment reduced odor related to cucumber and green descriptors, whilst significantly improved odors related to sweet, floral, and fruity descriptors, thus contributing to the overall flavor of watermelon juice. Compared with untreated watermelon juice, the amount and concentration of volatile compounds in ultrasonicated watermelon juice increased by 82.50% and 111.84%, respectively. Notably, 22 alkene compounds were newly formed in ultrasonicated watermelon juice, which contributed to sweet and fruity aroma of watermelon juice. The findings of the present study suggest that ultrasonic treatment may be a potential method to improve the overall flavor of watermelon juice.

## Highlights

• The optimal conditions for ultrasonic treatment were 325 W, 20 min.
• Ultrasonic treatment enhances the fruity, sweet and floral of watermelon juice.
• Ten key aroma-active compounds identified in ultrasonically treated watermelon juice.
• Alkene compounds can be used as discriminant index of ultrasonic treatment.

## 1 Introduction

Watermelon juice has gained popularity among consumers due to its unique flavor, attractive color, and potential health benefits [1]. Flavor is the most important quality attribute of watermelon juice. Generally, flavor of watermelon juice is characterized by green, cucumber, sweet and fruity odors. Odorants identified in watermelon juice are mainly C_6_ and C_9_ alcohols and aldehydes, such as hexanal, nonanal, (*E*,*Z*)-2,6-nonadienal, (*E*)-2-nonenal, (*Z*,*Z*)-3,6-nonadienal, (*Z*)-6-nonenol and (*E*,*Z*)-2,6-nonadienol [2], [3]. Among aroma compounds, (*E*,*Z*)-2,6-nonadienal is known as the cucumber aldehyde due to its strong cucumber aroma, whose OAV was 8282 in fresh watermelon juice [4]. In contrast, (*Z*,*Z*)-3,6-nonadienol is reportedly the aroma compound that most contributes to the overall aroma of watermelon juice, being thus known as the watermelon aroma [5]. In addition, (*E*)-2-nonenal is an important aroma compound in watermelon juice owing to its high concentration and low threshold value [6]. Flavor of watermelon juice is bland, which limits its development. Hence, it is necessary to develop an efficient processing technology to improve the overall flavor of watermelon juice.

Ultrasound has emerged as a new green technology in the food industry, which improves food processing efficiency without leading to contamination, thus having a great potential for wider application in the food industry [7]. Ultrasonic waves have a frequency higher than 20 KHz, and can be classified as low-intensity (<1 W/cm^2^) or high-intensity (10–1000 W/cm^2^) ultrasonic waves [8]. Ultrasound releases enormous amounts of energy in a liquid by generating bubbles through cavitation, which subsequently collapse, thus inducing mechanical, chemical, and biochemical changes in liquid properties [9]. In this context, ultrasonic cavitation affected the microstructure, quality attributes and physicochemical properties of treated foods.

In recent years, the use of ultrasonic waves has been largely studied as a means to improve physicochemical properties and sensory attributes of foods. It has been shown that ultrasonication led to an increase of 10% in odorants in debittered grapefruit juice, in particular the concentration of aroma compounds such as *d*-carvone, ethyl butyrate, ethyl 3-hydroxyhexanoate, and (*R*)-limonene [10]. In addition, ultrasonic treatment has been shown to improve taste, color, flavor, and mouthfeel of hog plum juice, which contributed also to improve slightly its overall acceptability [11]. Moreover, ultrasonic treatment could promote the release of a greater variety of key glycosidic aroma compounds in orange juice, including olefins, esters, and aldehydes [12]. In addition, ultrasonic treatment has been widely used to improve the sensory quality of several food products, including soybean milk [13], apple juice [14], fried meatballs [7], and salted egg yolk [15]. Furthermore, ultrasonic treatment could significantly improve the overall aroma and acceptability of red watermelon juice [16]. However, the effect of ultrasonic treatment on key aroma-active compounds in watermelon juice has not yet been investigated, and only scant information is available on the flavor of ultrasonicated watermelon juice.

Therefore, this study aimed to explore the effects of ultrasonic treatment onto watermelon juice using a molecular sensory approach. In particular, the following aspects of ultrasonicated watermelon juice were evaluated: i) determination of sensory attributes and volatile compounds in by sensory evaluation and GC-O-MS; ii) identification of aroma-active compounds by AEDA and OAV; and iii) validation of key aroma-active compounds by aroma recombination and omission experiment. This study provides a novel approach for improving flavor and processing of watermelon juice.

## 2 Materials and methods

### 2.1 Chemicals and reagents

Chemicals used in the study had purity > 99%. Citric acid, *n*-hexane, and glucose were purchased from Lab Gou e-mall (Beijing, China). Other chemicals, including (*Z*)-2-hexenal, (*Z*)-6-nonenal, *α*-pinene, nonanol, (*Z*)-6-noneneol, *β*-ionone, 2-methyl-3-heptanone, and *n*-alkanes (C_7_–C_30_) were purchased from Sigma-Aldrich (St. Louis, USA). Isovaleraldehyde, hexanal, (+)-limonene, 6-methyl-5-hepten-2-one, hexanol, nonanal, (*E*)-2-nonenal, (*E*,*Z*)-2,6-nonadienal, *β*-caryophyllene, (*Z*)-3-nonenol, (*E*,*Z*)-2,6-nonadienol, (*E*,*Z*)-3,6-nonadienol, and geranyl acetone, were purchased from TCI (Shanghai, China). Nitrogen gas (99.9992% purity) was purchased from Beijing AP BAIF Gases Industry Co., ltd. (Beijing, China). Liquid nitrogen was purchased from XianHeyu Trading Co., ltd. (Beijing, China).

### 2.2 Sample preparation

In order to eliminate individual differences, watermelon fruit units were obtained concurrently to ensure comparable maturity state and overall fruit quality. Twenty fruit units of watermelon Qilin variety, seedless, weighing approximately 2.5 kg each, planted in Panggezhuang, Daxing District, Beijing, China, were purchased at Yonghui Supermarket in Beijing, China. After surface hygienization and peeling, watermelon units were processed with a juicer, and the resulting liquid was then filtered using a gauze until foam was no longer observed, being thus considered as watermelon juice; FW samples were immediately submitted to the subsequent analysis.

### 2.3 Ultrasonic treatment conditions

Watermelon juice samples were sonicated using a probe ultrasonic processor (JY92-IIN; Ningbo Scientz Biotechnology Co., Ningbo, China) with a 25 KHz transducer and horn diameter of 6 mm. The main effects of various factors of ultrasonic treatment on the flavor of watermelon juice were tested with a single-factor experimental design. The ultrasonic power was: 65, 130, 195, 260, 325, 390 and 455 W; and ultrasonic times of 10, 20, 30, 40 and 50 min. The temperature was maintained below 40 °C in ice-water bath, and the pulse mode was used for 2 s in duty cycle (10 s in activity, 2 s in inactivity). Samples were analyzed within 12 h after juice preparation, and three independent experiments were conducted.

### 2.4 Sensory analysis

Aroma profile of watermelon juices was evaluated by twelve panelists (six males and six females; average age of panelists: 25 years old); each panelist had at least one-year’s experience practicing sensory attributes relevant to the experiment, ensuring that panelists would be able to accurately identify aromas of watermelon juice. Flavor profiles were described, including overall flavor, cucumber, green, fruity, sweet, and floral. In particular, six odor attributes were similar to the smell of watermelon, cucumber, watermelon rind, mixed fruit, mixed candy, and flowers, respectively. Samples were randomly assigned a three-digit code and then presented to each panelist in random order for odor perception evaluation. An evaluation scale of 0–5 points with 0.5 incremental point scale was used in the analysis. The necessary sensory recovery rest (1 min) was given between each sample. Coffee beans were provided for olfactory recovery. Each sample was analyzed in triplicate.

### 2.5 Solid-phase microextraction (SPME) of volatile compounds

Volatile compounds from watermelon juice were extracted using SPME in a 50/30-μm DVB/CAR/PDMS fiber with column length of 2 cm (Supelco, Bellefonte, PA, USA) [17]. Briefly, 10 mL of watermelon juice and 1 μL of 2-methyl-3-heptanone (0.816 μg/μL, internal standard) were transferred into a 40 mL glass vial with a silicon septum. The sample was placed in a thermostatic water bath and equilibrated for 20 min at 40 °C. The fiber was exposed to the sample headspace for 40 min at 40 °C. After volatile compound extraction, the SPME fiber was inserted into the GC injector for thermal desorption at 250 °C for 5 min. Each sample was analyzed in triplicate.

### 2.6 Gas chromatography–olfactometry-mass spectrometry (GC-O-MS) analysis

An Agilent 7890A-5977 GC–MS system (Agilent Technologies Inc., Santa Clara, CA, USA) was used to identify volatile compounds. A polar DB-WAX capillary column (60 mm × 0.25 mm, 0.25-μm film thickness; J & W Scientific, Folsom, CA, USA) was used to separate volatile compounds. The initial column temperature was set at 40 °C, followed by a 5 min-hold, and a subsequent increase to 190 °C at the rate of 3 °C/min. The final reached temperature was 230 °C at the rate of 8 °C/min, which was maintained for 15 min. Ultrapure helium was used as the carrier gas. Electron-impact mass spectra were generated at 70 eV ionization energy within the *m*/*z* scan range of 50–350. The temperature of MS source and quadrupole was set at 230 °C and 150 °C, respectively.

An Agilent 7890A series GC combined with an olfactory detection port (Sniffer 9000, Brechbühler, Schlieren, Switzerland) was used to discriminate aroma-active compounds in watermelon juice. The temperature of the olfactory port and transfer line was kept at 230 °C and 250 °C, respectively. GC-O analyses were performed by three well-trained panelists. Prior to experiments, panelists enrolled in the experiment trained for at least one month by smelling reference odor compounds at different concentrations in model solutions for 2 h daily. During GC-O analysis, the wet gas was delivered to the nose via a blank capillary column for improving the sensitivity of panelists. Perceived aroma intensity and RT were recorded by the panelists [18]. If two or more panelists detected the aroma, the respective aroma-active compound was considered as present in the sample.

### 2.7 Aroma extraction dilution analysis (AEDA)

AEDA was used to characterize the contribution of perceived odorants to the overall aroma of watermelon juice samples. A 3*^n^*:1 serial dilution was performed by varying the split ratio of GC inlet [19]. Sensory evaluation was performed by GC-O based on the evaluation of three experienced panelists (two females and one male) for each diluted until no aroma was perceived.

### 2.8 Qualitative analysis of aroma-active compounds

Volatile compounds in watermelon juice samples were analyzed qualitatively following the method of Yang et al. with minor modifications [20]. Volatile compounds were identified by comparing mass spectrometry data with the NIST 17 Mass Spectral Library. A series of *n*-alkanes (C_7_-C_30_) was used to determine the RI of each volatile compound. Subsequently, identified compounds were reconfirmed by comparing calculated value with RI reported in literature. The actual smell results were compared with theoretical odor of target compounds, and results were verified using standard odor compounds.

### 2.9 Quantitative analysis of aroma-active compounds

The external standard method was used to quantify aroma-active compounds in watermelon juice samples [19]. Standard solutions of aroma-active compounds in FW and ultrasonicated watermelon juice (UW) samples are shown in Table 2. The selective ion mode (SIM) was used for accurate quantification of aroma-active compounds. A mixed stock solution was prepared by 1000-fold dilution of original authentic standard odor compounds in absolute *n*-hexane, which were then serially diluted to seven concentrations (Supplement 1). Each standard of mixed stock solutions was spiked with 2-methyl-3-heptanone (0.816 μg/μL) as an internal standard. Three groups of mixed stock solutions were prepared for GC analysis at different range-level responses: the first group included 2-hexenal, hexanol, and *β*-ionone; the second group contained 3-methylbutanal, hexanal, limonene, 6-methyl-5-hepten-2-one, (*Z*)-6-nonenol and geranyl acetone; the three group was constituted by nonanal, (*Z*)-6-nonenal, α-pinene, (*E*)-2-nonenal, (*Z*)-3-nonenol, (*E*,*Z*)-2,6-nonadienal, *β*-caryophyllene, nonanol, (*E*,*Z*)-3,6-nonadienol, (*E*,*Z*)-2,6-nonadienol. Standard curves were obtained by comparing the response ratios of standard odor compounds and internal standards to their respective concentrations (Table 2).

### 2.10 Determination of odor activity values (OAVs)

OAVs were calculated as the ratio of the concentration of each aroma-active compound to its respective threshold in water. In addition, the threshold of different compounds was stipulated based on information reported in previous studies [21].

### 2.11 Aroma recombination and omission experiment

To confirm aroma-active compounds identified by AEDA and OAVs, aroma reconstitution models were prepared to compare UW aroma profile with FW aroma profile of watermelon juice samples [22]. A model was prepared in ultrapure water containing 5% fructose and organic acids, and 0.30% pectin to simulate the watermelon juice matrix [23]. Two synthetic aroma solutions were prepared as follows: the aroma recombination model 1 included fourteen major aroma-active compounds identified in FW (chosen based on FD factor ≥ 3 and OAV ≥ 1; Table 1 and Table 2); the aroma recombination model 2 included sixteen major aroma-active compounds identified in UW (chosen based on FD factor ≥ 3 and OAV ≥ 1; Table 1 and Table 2).

**Table 1:** The volatile compounds in FW and UW samples.

NO.	Compounds	Aroma attributes a	RI b	Concentration (μg/L) c		Means of identification d	FD factor e	
				FW	UW		FW	UW
Aldehydes								
1	ethanal	pungent, ether	811	49.153 ± 0.267^a^	9.489 ± 0.582^b^	MS/RI	–	–
2	butanal	pungent	874	3.884 ± 0.210^a^	–	MS/RI	–	–
3	2-methylbutanal	cocoa, almond	898	40.737 ± 0.221^a^	2.938 ± 0.213^b^	MS/RI	–	–
4	3-methylbutanal	malt	902	34.357 ± 0.187^a^	1.493 ± 0.135^b^	MS/RI/O/STD	3	–
5	2,4-hexadienal	green, sweet	936	–	0.711 ± 0.018^a^	MS/RI/O	–	–
6	pentanal	almond, malt	964	1.675 ± 0.090^a^	0.808 ± 0.066^b^	MS/RI	–	–
7	hexanal	grass	1071	65.994 ± 0.358^a^	19.054 ± 0.103^b^	MS/RI/O/STD	9	9
8	(*E*)-2-pentenal	strawberry, fruit	1236	–	1.581 ± 0.149^a^	MS/RI	–	–
9	heptanal	fat	1279	2.063 ± 0.110^b^	5.129 ± 0.327^a^	MS/RI	–	–
10	2-hexenal	green, apple	1311	6.152 ± 0.330^b^	12.877 ± 0.070^a^	MS/RI/O/STD	3	3
11	octanal	green, lemon	1382	1.342 ± 0.070^b^	4.171 ± 0.093^a^	MS/RI	–	–
12	(*E*)-2-heptenal	fat	1419	–	2.242 ± 0.083^a^	MS/RI	–	–
13	2,6-dimethyl-5-heptenal	green, melon	1448	–	2.316 ± 0.444^a^	MS/RI	–	–
14	nonanal	green	1485	105.946 ± 2.201^a^	67.898 ± 1.073^b^	MS/RI/O/STD	9	9
15	(*E*)-2-octenal	green, nut	1526	–	5.464 ± 0.252^a^	MS/RI	–	–
16	(*E*)-4-nonenal	fruity	1533	–	5.624 ± 0.101^a^	MS/RI	–	–
17	(*Z*)-6-nonenal	green, cucumber	1547	109.934 ± 0.108^b^	178.938 ± 3.862^a^	MS/RI/O/STD	81	81
18	benzaldehyde	almond, burnt sugar	1619	–	20.057 ± 0.109^a^	MS/RI	–	–
19	(*E*)-2-nonenal	green, cucumber	1636	414.885 ± 2.253^a^	288.072 ± 1.565^b^	MS/RI/O/STD	81	81
20	(*E*,*Z*)-2,6-nonadienal	green, cucumber	1685	101.393 ± 0.551^a^	83.015 ± 0.733^b^	MS/RI/O/STD	243	81
21	(*E*,*E*)-2,4-nonadienal	green	1719	–	0.632 ± 0.074^a^	MS/RI	–	–
22	*β*-cyclocitral	lemon	1723	3.191 ± 0.170^a^	–	MS/RI/O	–	–
23	(*E*)-cinnamaldehyde	cinnamon	2069	–	482.153 ± 13.482^a^	MS/RI	–	–
24	(*E*)-2-methoxycinnamaldehyde	sweet	2331	–	102.048 ± 0.695^a^	MS/RI	–	–
Concentration of total aldehydes				940.707 ± 6.246^b^	1296.710 ± 21.54^a^			

Alcohols								
25	ethanol	sweet	923	–	0.953 ± 0.076^a^	MS/RI/O	–	–
26	1-penten-3-ol	butter, sweet	1267	2.962 ± 0.016^a^	0.590 ± 0.017^b^	MS/RI/O	–	–
27	1,8-cineole	mint, sweet	1302	–	1.300 ± 0.021^a^	MS/RI	–	–
28	2-methybutanol	wine, onion	1305	5.582 ± 0.030^a^	0.531 ± 0.003^b^	MS/RI	–	–
29	pentanol	balsamic	1349	4.515 ± 0.250^a^	3.113 ± 0.066^b^	MS/RI/O	–	–
30	(*Z*)-2-pentenol	green	1416	1.864 ± 0.010^a^	–	MS/RI/O	–	–
31	(*E*)-2-pentenol	mushroom	1417	–	1.388 ± 0.078^a^	MS/RI/O	–	–
32	hexanol	green, flower	1451	30.349 ± 0.165^a^	17.708 ± 0.186^b^	MS/RI/O/STD	3	3
33	(*Z*)-3-hexenol	grass	1481	11.507 ± 0.620^a^	12.021 ± 0.770^a^	MS/RI	–	–
34	2-ethylhexanol	green, rose	1587	6.180 ± 0.034^a^	–	MS/RI	–	–
35	octanol	green	1656	5.765 ± 0.310^a^	–	MS/RI/O	–	–
36	(*E*)-2-octenol	soap, plastic	1712	4.756 ± 0.260^a^	–	MS/RI	–	–
37	nonanol	green	1761	130.152 ± 0.707^b^	220.195 ± 6.831^a^	MS/RI/O/STD	3	27
38	(*Z*)-3-nonenol	green, cucumber	1788	989.054 ± 5.372^b^	1066.914 ± 23.685^a^	MS/RI/O/STD	9	3
39	(*E*)-2-nonenol	green, melon	1810	88.442 ± 0.480^b^	137.105 ± 0.908^a^	MS/RI/O/STD	3	3
40	(*Z*)-6-nonenol	green, cucumber	1816	31.970 ± 0.174^a^	–	MS/RI/O/STD	27	–
41	(*E*,*Z*)-3,6-nonadienol	green, cucumber	1842	281.526 ± 1.529^b^	351.621 ± 10.040^a^	MS/RI/O/STD	9	27
42	(*E*,*Z*)-2,6-nonadienol	green, cucumber	1849	128.012 ± 0.152^b^	199.596 ± 5.616^a^	MS/RI/O/STD	81	81
43	(−)-cubenol	green tea	2089	–	36.141 ± 0.901^a^	MS/RI	–	–
44	*T*-muurolol	herb	2162	–	20.999 ± 0.114^a^	MS/RI	–	–
45	*α*-cadinol	herb, wood	2198	–	13.535 ± 0.426^a^	MS/RI	–	–
46	cinnamyl alcohol	oil	2207	–	14.353 ± 0.782^a^	MS/RI	–	–
Concentration of total alcohols				1722.636 ± 8.813^b^	2098.066 ± 50.147^a^			

Ketones								
47	3-pentanone	ether	964	2.496 ± 0.140^a^	–	MS/RI	–	–
48	3-octanone	herb	1350	2.095 ± 0.110^a^	–	MS/RI	–	–
49	3-hydroxy-2-butanone	butter, cream	1380	1.048 ± 0.060^a^	–	MS/RI/O	–	–
50	6-methyl-5-hepten-2-one	mushroom	1432	25.287 ± 0.137^b^	55.579 ± 1.006^a^	MS/RI/O/STD	9	–
51	geranyl acetone	fruity	1926	13.492 ± 0.730^b^	47.080 ± 0.256^a^	MS/RI/O/STD	27	27
52	*β*-ionone	green	1996	–	9.789 ± 0.758^a^	MS/RI/O/STD	–	3
53	coumarin	green, sweet	2326	–	17.552 ± 1.313^a^	MS/RI	–	–
Concentration of total ketones				44.418 ± 0.241^b^	130.001 ± 0.706^a^			

Alkenes								
54	α-pinene	pine	1012	–	0.771 ± 0.024^a^	MS/RI	–	–
55	camphene	camphor	1054	–	0.825 ± 0.066^a^	MS/RI	–	–
56	(−)-*β*-pinene	green	1089	–	1.830 ± 0.080^a^	MS/RI	–	–
57	α-phellandrene	mint	1257	–	1.278 ± 0.077^a^	MS/RI	–	–
58	limonene	lemon, orange	1286	–	55.232 ± 0.099^a^	MS/RI/O/STD	–	3
59	phenylethylene	balsamic	1347	–	5.834 ± 0.173^a^	MS/RI	–	–
60	terpinolene	pine, plastic	1374	–	1.456 ± 0.666^a^	MS/RI	–	–
61	*α*-cubebene	herb, wax	1561	–	6.755 ± 0.175^a^	MS/RI	–	–
62	*α*-guaiene	wood, balsamic	1590	–	61.121 ± 1.036^a^	MS/RI/O	–	–
63	*α*-copaene	honey, sweet	1609	–	663.038 ± 41.044^a^	MS/RI/O/STD	–	9
64	(+)-longifolene	sweet, flower	1644	–	120.120 ± 1.357^a^	MS/RI/O	–	–
65	aromadendrene	citrus, fruity	1658	–	36.037 ± 0.900^a^	MS/RI/O/STD	–	9
66	*β*-elemene	sweet	1695	–	94.906 ± 1.220^a^	MS/RI	–	–
67	*β*-caryophyllene	sweet	1707	–	265.687 ± 4.965^a^	MS/RI/O/STD	–	27
68	*α*-humulene	wood	1779	–	204.630 ± 1.816^a^	MS/RI	–	–
69	*γ*-muurolene	herb	1800	–	54.859 ± 3.555^a^	MS/RI	–	–
70	*α*-muurolene	wood	1827	–	77.344 ± 0.709^a^	MS/RI	–	–
71	*γ*-cadinene	wood	1858	–	139.465 ± 1.462^a^	MS/RI	–	–
72	*α*-curcumene	herb	1862	–	52.700 ± 1.695^a^	MS/RI	–	–
73	cadinadiene	fruity	1875	–	182.705 ± 1.697^a^	MS/RI/O/STD	–	3
74	calamenene	herb	1914	–	44.170 ± 0.977^a^	MS/RI	–	–
75	*α*-calacorene	wood	1978	–	99.175 ± 1.243^a^	MS/RI	–	–
Concentration of total alkenes				–	2169.939 ± 63.088^a^			

Heterocycles								
76	2-methylfuran	chocolate	885	2.370 ± 0.130^a^	1.505 ± 0.022^b^	MS/RI	–	–
77	2,5-dimethylfuran	meaty	937	1.196 ± 0.060^a^	–	MS/RI	–	–
78	2-amylfuran	green bean, butter	1319	11.481 ± 0.620^b^	69.122 ± 0.728^a^	MS/RI	–	–
79	(*E*)-2-(pent-1-en-1-yl)furan	roasted	1392	–	4.828 ± 0.097^a^	MS/RI	–	–
Concentration of total heterocycles				15.047 ± 0.082^b^	75.455 ± 0.847^a^			

Aromatic								
80	toluene	paint	1028	2.699 ± 0.150^b^	5.339 ± 0.192^a^	MS/RI	–	–
81	1,2-dimethylbenzene	geranium	1239	–	0.575 ± 0.024^a^	MS/RI	–	–
82	4-isopropyltoluene	gasoline	1360	0.446 ± 0.020^b^	2.774 ± 0.086^a^	MS/RI	–	–
83	2,3-dimethylnaphthalene	earthy	2043	–	1.449 ± 0.029^a^	MS/RI	–	–
Concentration of total aromatic				3.144 ± 0.017^b^	10.137 ± 0.331^a^			

Others								
84	ethyl acetate	pineapple	881	–	1.249 ± 0.014^a^	MS/RI/O	–	–
85	nonanoic acid	sour	2170	3.299 ± 0.018^a^	–	MS/RI	–	–
Concentration of other compounds				3.299 ± 0.018^a^	1.249 ± 0.014^b^			

The different letters marked with a and b on the compound concentrations represent significant differences between the data, *Sig* < 0.05, and there is no significant difference between the data marked with the same letters.

a The aroma description of each volatile compounds that sensed on sniffer port of GC-O in FW and UW samples. In addition, if a compound could not be detected by sniffer, it's aroma attribute was cited from references.

b The actual retention index on Capillary DB-WAX.

c Mean values of triplicates with standard deviations (SD).

d The different identification method including MS, RI, O, and STD.

e The FD factors of all aroma compounds in samples calculated by varying the split ratio of GC inlet.

**Table 2:** Concentrations and odor activity values of aroma-active compounds in FW and UW samples.

Aroma-active compounds^a^	Odor threshold in water (μg/L) ^b^	Concentration (μg/L) ^c^		OAV^d^		Linear equations^e^	R^2^	Concentration range^f^	Quota selected ion (*m*/*z*)
		FW	UW	FW	UW				
*β*-ionone	0.007	–	22.723 ± 0.032^a^	–	3249	y = 0.5897x + 0.0194	0.9946	2.33–466.00	177, 43, 192
(*E*,*Z*)-2,6-nonadienal	0.11	388.935 ± 0.050^a^	343.338 ± 0.054^b^	3536	3122	y = 0.243x + 0.0041	0.9986	43.00–860.00	41, 70, 123
nonanal	1.1	284.836 ± 0.051^b^	387.051 ± 0.073^a^	259	352	y = 0.1982x + 0.0063	0.9948	41.35–827.00	57, 98, 124
(*Z*)-6-nonenal	1	180.950 ± 0.070^b^	229.211 ± 0.298^a^	181	229	y = 0.1953x + 0.0037	0.9968	41.70–834.00	41, 57, 122
(*E*)-2-nonenal	1.5	201.181 ± 0.027^b^	260.138 ± 0.054^a^	134	173	y = 0.1596x + 0.0041	0.9966	41.70–834.00	43, 70, 111
(*E*,*Z*)-2,6-nonadienol	1	143.891 ± 0.013^b^	165.018 ± 0.025^a^	144	165	y = 0.2839x + 0.0088	0.9924	43.00–860.00	41, 93, 122
3-methylbutanal	0.35	41.696 ± 0.006^b^	47.045 ± 0.064^a^	119	135	y = 0.3022x + 0.0074	0.9974	8.00–400.00	44, 58, 86
(*Z*)-6-nonenol	1	24.332 ± 0.030^a^	–	24	–	y = 0.6292x + 0.0055	0.9931	8.45–422.50	67, 95, 124
nonanol	18	144.977 ± 0.004^b^	152.126 ± 0.037^a^	8	8	y = 0.5463x + 0.0166	0.9906	41.35–827.00	56, 70, 97
hexanal	5	39.731 ± 0.044^a^	27.884 ± 0.119^b^	8	6	y = 0.7749x + 0.0054	0.9914	8.01–400.50	56, 44, 82
2-hexenal	4	27.670 ± 0.029^a^	25.941 ± 0.058^a^	7	6	y = 0.1103x − 0.0011	0.9935	2.07–414.00	41,69,98
6-methyl-5-hepten-2-one	18.89	111.666 ± 0.023^a^	–	6	–	y = 0.5284x + 0.0095	0.9992	8.55–427.50	43, 108, 126
hexanol	5.6	9.412 ± 0.017^b^	23.021 ± 0.030^a^	2	4	y = 0.5947x + 0.0041	0.9995	2.17–408.50	56, 43, 84
*α*-copaene	100	–	404.146 ± 0.065^a^	–	4	y = 0.0717x + 0.0016	0.9966	44.95–899.00	161, 119, 204
*β*-caryophyllene	64	–	211.219 ± 0.310^a^	–	3	y = 0.685x − 0.0001	0.9989	45.05–901.00	93, 133, 204
limonene	34	–	83.711 ± 0.015^a^	–	2	y = 0.9118x − 0.0081	0.9993	8.42–421.00	68, 93, 136
(*E*,*Z*)-3,6-nonadienol	200	229.725 ± 0.036^b^	242.022 ± 0.031^a^	1	1	y = 0.8505x + 0.0074	0.9994	43.00–860.00	67, 93, 140
geranyl acetone	60	65.847 ± 0.009^b^	89.890 ± 0.127^a^	1	1	y = 0.2407x + 0.0052	0.9940	8.73–436.50	43, 69, 151
(*Z*)-3-nonenol	70,000	100.406 ± 0.008^b^	158.872 ± 0.040^a^	<1	<1	y = 0.2986x + 0.0072	0.9951	42.25–845.00	55, 81, 124

^a^Volatile compounds that FD > 3, except no peak compounds.

^b^Odor thresholds were referenced as a book, named: odor thresholds compilations of odor threshold values in air, water and other media (second enlarged and revised edition).

^c^Average of three replicates.

^d^OAVs were calculated by dividing the concentrations by the odor thresholds.

^e^Linear equations were fitted by peak area and corresponding concentration in samples.

^f^The applicable concentration range of the linear equation.

^g^The largest molecular weight was the mother ion, and the rest of ions was for quantitative.

The different letters marked with a and b on the compound concentrations represent significant differences between the data, *Sig* < 0.05, and there is no significant difference between the data marked with the same letters.

Different mixture models were prepared by omitting one aroma-active compound at a time among major aroma-active compounds identified in watermelon juice samples. The differences between each omission model and the complete reconstituted model were compared using the triangle test methodology according to the Chinese Standards GB/T 12311–2012 [24]. Briefly, if 8, 9, and 10 of the 12 panelists could identify the difference when a compound was eliminated, the results were indicated as significant (*α* ≤ 0.05), highly significant (*α* ≤ 0.01), or very highly significant (α ≤ 0.001), respectively. If this number was < 8, the result was considered ‘not significant’.

### 2.12 Statistical analysis

All experiments were performed in triplicate, and data were expressed as mean ± standard deviation. OriginPro 2019b (OriginLab Corp., Washington, MA, USA) and Microsoft Office 2019 (Microsoft Corp., Redmond, WA, U.S.A.) were used for data analysis and graphs. IBM SPSS Statistics 26 (IBM, Chicago, USA) was used to independent-samples T Test (*Sig* ≤ 0.05) and significance analysis (*P* ≤ 0.05).

## 3 Results and discussion

### 3.1 Effect of ultrasonic treatment conditions on the release of volatile compounds

**Fig. 1:**
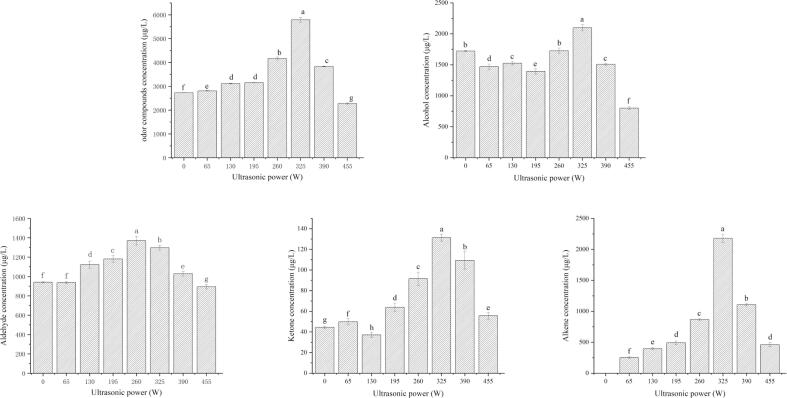
The effects of ultrasonic power on odor compounds in watermelon juice.

Fig. 1 shows the effect of ultrasonic power on the release of volatile compounds from watermelon juice. Volatile compounds detected from watermelon juice can be divided into alcohols, aldehydes, ketones, alkenes and other compounds. With the increase of ultrasonic power, the concentration of volatile compounds increased first and then decreased, and the highest concentration was at 325 W. Compared with the control group, the total concentration increased by 112.25%. The total volatile compound concentrations in the 65–390 W ultrasound treatment groups increased significantly compared with control groups (*P* < 0.05). This might be due to the increased level of lipid oxidation and amino acid degradation caused by low-frequency ultrasonic treatment as compounds form the flavor of watermelon juice mainly from lipid oxidation, amino acid degradation and others [3], [23]. And the concentration of volatile compounds was significantly reduced under 455 W ultrasound (*P* < 0.05). This may be the ultrasonic power is too high, the formation of cavitation bubbles increases, and the cavitation bubbles may become too large to break, or break very weakly, and too many bubbles are not conducive to the propagation of ultrasound, thus reducing the cavitation effect [12].

**Fig. 2:**
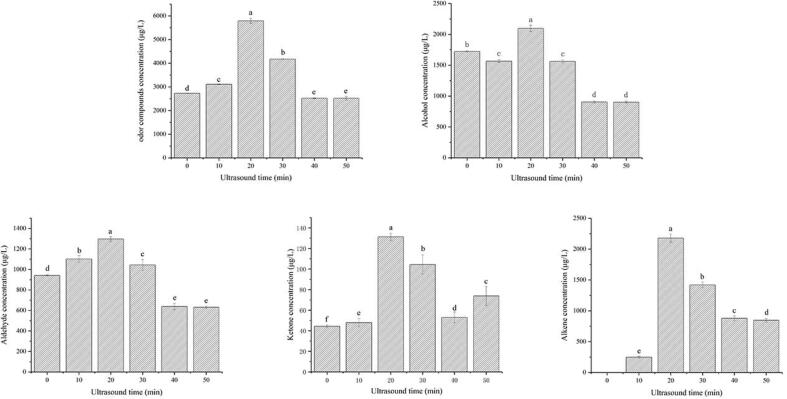
The effects of ultrasonic time on odor compounds in watermelon juice.

Fig. 2 shows the effect of ultrasonic time on the release of volatile compounds from watermelon juice. The highest total concentration of volatile compounds was found at 20 min of ultrasonic treatment, which is consistent with Guerrouj’s discovered that the sensory evaluation of orange juice is unacceptable after ultrasonic treatment for 20 min [25]. Therefore, the optimal conditions for ultrasonic treatment of watermelon juice are as follows: ultrasonic power 325 W, ultrasonic time 20 min.

### 3.2 Sensory analysis

**Fig. 3:**
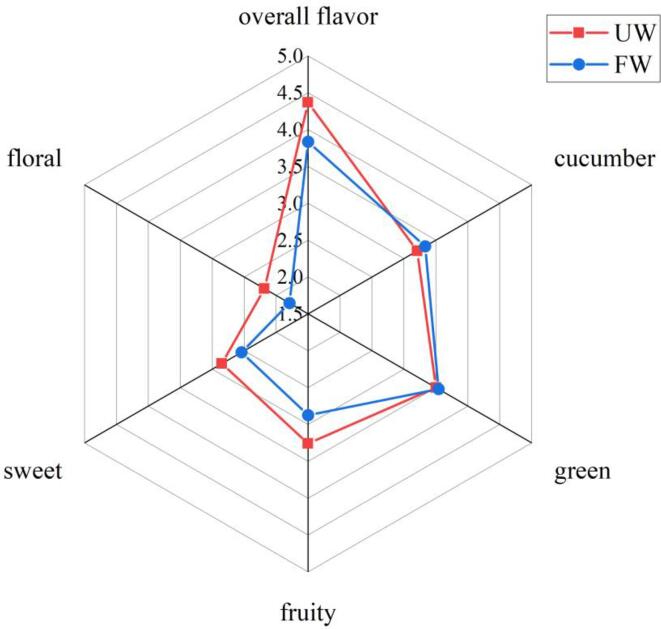
The result of sensory evaluation among FW and UW.

First, the aroma profiles of FW and UW were compared (Fig. 3). The aroma profiles of FW and UW were comparably similar, and green, cucumber, sweet and fruity aromas could be well perceived. After ultrasonic treatment, the overall flavor of watermelon juice was significantly improved (*Sig* < 0.05). Compared with FW, sensory scores of floral, fruity, and sweet odor attributes in UW were significantly improved by 21.79%, 13.19%, and 12.20%, respectively, which may have largely contributed to the differences in aroma of UW and FW samples. In particular, green and cucumber flavor attributes showed a downward trend in UW samples, although no significant difference was observed (*Sig* > 0.05). These findings were similar to those reported in a previous study that explored the improvement in sensory scores of hot plum juice treated by ultrasonication [11].

### 3.3 Identification of volatile compounds in FW and UW

**Fig. 4:**
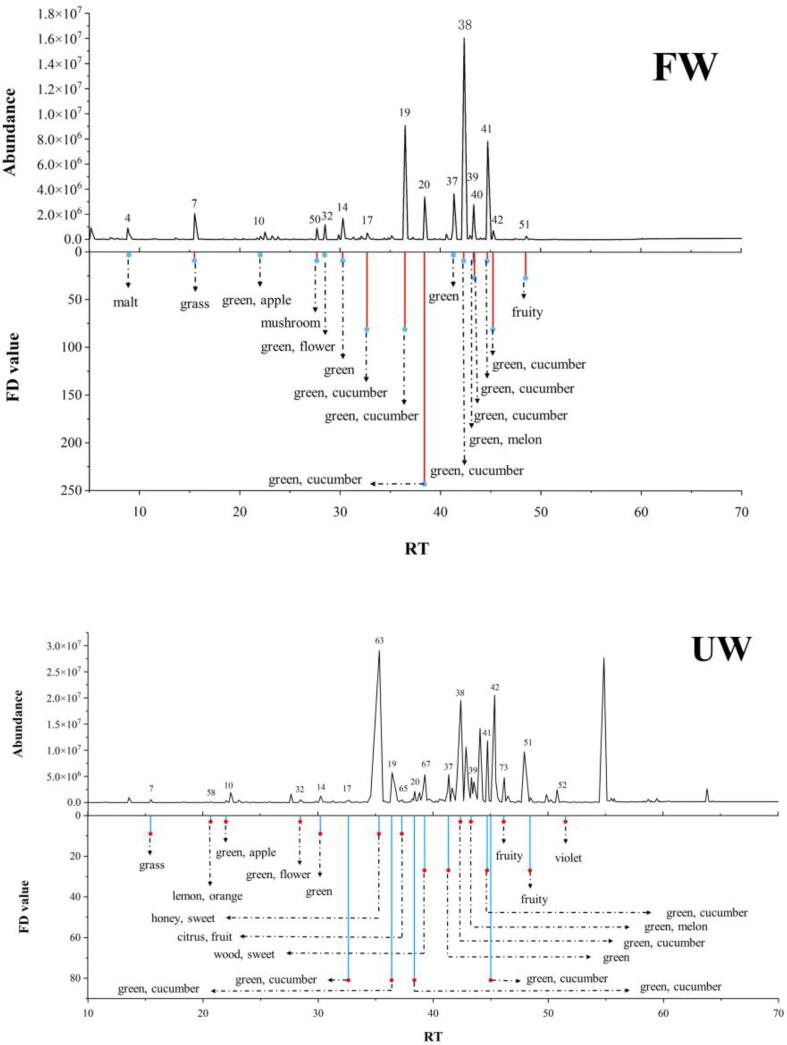
GC–MS spectra and key odor compounds (FD > 3) of FW and UW (The number was in accordance with that in Table 1).

The plots of GC–MS data of FW and UW samples are shown in Fig. 4. In total, 85 volatile compounds were identified in FW and UW (Table 1); 34 volatile compounds could be detected at the sniffing port and were considered as aroma-active compounds, which comprised mainly aldehydes, alcohols, ketones, and alkenes. Compared with FW, the types and concentrations of volatile compounds in UW samples were significantly increased (*Sig* < 0.05), and 22 alkene compounds were uniquely detected. The cavitation effects induced by ultrasonic treatment, including free radical formation, promoted polymerization/depolymerization, and other chemical reactions in UW samples, which may explain higher diversity and concentration of volatile compounds in UW samples [14].

The concentration of alcohol compounds in FW was 1722.64 μg/L; however, in UW samples, the concentration of such compounds was significantly increased (*Sig* < 0.05) 2098.07 μg/L. Alcohols in FW and UW were mainly C_6_ and C_9_ alcohols, (*E*,*Z*)-3,6-nonadienol, (*Z*)-3-nonenol, and nonanol. These alcohols are generally considered the products of oxidative degradation of polyunsaturated fatty acids such as linoleic acid and linolenic acid [26]. Free radicals generated by ultrasonication may promote autoxidation, and oxidation of unsaturated fatty acids may promote the formation of alcohols [13].

The concentration of aldehyde compounds in FW was 940.71 μg/L; in UW samples, the concentration of aldehyde compounds significantly increased (*Sig* < 0.05) (1296.71 μg/L). The concentration of (*E*)-2-nonenal, (*Z*)-6-nonenal, and nonanal was higher in FW, whereas the concentration of (*E*)-cinnamaldehyde, (*E*)-2-nonenal, and (*Z*)-6-nonenal was higher in UW. Early research studies described that aldehydes are the oxidation products of polyunsaturated fatty acids [23]. It has been shown that ultrasonic treatment promoted oxidation of unsaturated fatty acids, resulting in an increase of aldehydes [13]. In addition, under ultrasonication, alcohol compounds may also be oxidized to aldehydes [27].

The concentration of ketone compounds in FW was 44.42 μg/L; in contrast, the concentration of ketone compounds in UW significantly increased (*Sig* < 0.05) (130.00 μg/L). The concentration of 6-methyl-5-hepten-2-one and geranyl acetone was higher in FW, whereas the concentration of 6-methyl-5-hepten-2-one, geranyl acetone, and *β*-ionone was higher in UW. *β*-ionone is formed by the degradation of *β*,*β*-carotene [3]. Geranyl acetone is derived from phytoene, phytofluene or ζ-carotene [23]. In another study, ultrasonic treatment improved the release of lycopene and carotene from the juice [28]. With prolonged ultrasonic treatment, hydroxyl radicals and hydrogen atoms generated by cavitation promoted oxidative degradation of lycopene and carotenoids, and increased concentration of ketones [14].

The concentration of alkenes compounds in UW was 2169.94 μg/L, which was the highest concentration of all types of volatile compounds in UW, and all of them were newly formed after ultrasonic treatment of watermelon juice. No alkenes compounds were detected in FW, which may be due to their presence in the form of glycosides-bonded aroma substances with terpenes as ligands [12]. Cavitation induced by ultrasonic waves increases the agitation of the medium thus forming bubbles, which subsequently burst, leading to the disruption of glycosidic bonds and the release of olefinic compounds [29]. The findings observed herein are consistent with previous studies which used ultrasonic-assisted extraction to improve the recovery rate of terpenes in spearmint [30].

### 3.4 Identification of aroma-active compounds in FW and UW samples

AEDA method was used to characterize the contribution of aroma-active compounds to the overall aroma of watermelon juice. A total of 22 major aroma-active compounds with FD factors ≥ 3, which were classified into four groups (i.e., aldehydes, alcohols, ketones, and alkenes), were tentatively identified in FW and UW samples (Table 1, Fig. 4); 16 compounds with higher odor intensity were found in FW samples, whereas 19 compounds with higher odor intensity were found in UW.

Seven aldehydes with high FD values were detected in FW, namely (*E*,*Z*)-2,6-nonadienal (cucumber, FD = 243), (*E*)-2-nonenal (cucumber, FD = 81), (*Z*)-6-nonenal (cucumber, FD = 81), nonanal (green, FD = 9), hexanal (grass, FD = 9), 2-hexenal (apple, FD = 3), and 3-methylbutanal (malt, FD = 3), which were related to green and cucumber sensory attributes. Among these, (*E*,*Z*)-2,6-nonadienal, (*E*)-2-nonenal, and (*Z*)-6-nonenal have been reported as having strong odor properties in watermelon juice; in addition, C_6_ and C_9_ saturated aldehydes, as well as alkenes, have been reported as the main flavor compounds in watermelon juice [6], [31]. In UW, FD factors of 3-methylbutanal and (*E*,*Z*)-2,6-nonadienal decreased, whereas AEDA results of other aldehydes remained unchanged. These alterations were consistent with the results of sensory analysis of UW and FW, which revealed that cucumber odor is weaker in UW than in FW.

Seven alcohols with high FD values were detected in FW, namely (*E*,*Z*)-2,6-nonadienol (cucumber, FD = 81), (*Z*)-6-nonenol (cucumber, FD = 27), (*E*,*Z*)-3, 6-nonadienol (cucumber, FD = 9), (*Z*)-3-nonenol (cucumber, FD = 9), nonanol (green, FD = 3), hexanol (flower, FD = 3), and (*E*)-2-nonenol (melon, FD = 3). These results were in accordance with previous studies which indicated that C_9_ alcohols play a key role in green, melon, and cucumber aroma formation in watermelon juice [6], [23], [32]. Interestingly, (*Z*)-6-nonenol (cucumber, FD = 27), was uniquely detected in FW.

Two ketones with high FD values were detected in FW, including geranyl acetone (fruity, FD = 27) and 6-methyl-5-hepten-2-one (mushroom, FD = 9). Geranyl acetone was detected in FW and UW, which was the key aroma compound in watermelon juice. In addition, 6-methyl-5-hepten-2-one with mushroom odor in fresh watermelon juice is considered an off-flavor compound [6]. *β*-ionone (green, FD = 3) was perceived in UW, thus giving the sample a green and floral flavor.

Five alkenes with high FD values were detected in UW, including *β*-caryophyllene (sweet, FD = 27), α-copaene (sweet, FD = 9), aromadendrene (fruity, FD = 9), limonene (lemon, FD = 3) and cadinadiene (fruity, FD = 3), which conferred sweet and fruity sensory attributes. Among these, limonene has a faint citric aroma, being also known as the highest aroma-active compound in orange juice. Similarly, α-copaene and aromadendrene also confer ‘fruity’ and ‘sweet’ odors. Many studies have reported the presence and the contribution of these alkenes to the overall aroma of citrus fruits [33], [34]. Thus, it can be stated that the presence of these alkene compounds enriched the flavor of UW, and conferred desirable sweet and fruity aroma, and could be considered organic volatile markers to distinguish ultrasonicated and fresh watermelon juice.

### 3.5 Quantification of aroma-active compounds and OAVs

The results of the quantification of aroma-active compounds (FD ≥ 3) in FW and UW as determined by the external standard method are shown in Table 2. In contrast to FW, α-copaene, *β*-caryophyllene and limonene were found at higher concentrations in UW. In addition, the concentration of other aroma-active compounds changed significantly in UW samples. Moreover, the concentration of nonanal and (*E*,*Z*)-2,6-nonadienal was the highest in FW, which was similar to findings reported in previous studies [6].

Rather than the concentrations of the compounds, OAV determines the contribution of aroma-active compounds to the overall aroma profile of a sample. Basically, compounds with OAVs > 1 are considered aroma-active [35]. Therefore, OAV calculations were conducted in the present study to determine the contributions of aroma-active compounds to the aroma profile of FW and UW (Table 2).

In total, 17 compounds with OAV > 1 were found in UW, which were identified herein as major aroma-active compounds in UW, thus contributing to its aroma profile. *α*-copaene, *β*-caryophyllene, limonene, and *β*-ionone were uniquely detected in UW, which corroborates AEDA data; thus, it can be considered that these four compounds were the major aroma-active compounds that improved fruity and sweet taste of UW. In addition, (*E*,*Z*)-2,6-nonadienal showed the highest OAV in FW, but decreased in UW. Although AEDA data indicated that certain aroma-active compounds had high FD factors, their odor thresholds in water were relatively high, which results in low OAV [19]. For instance, (*Z*)-3-nonenol had high FD factor but OAV < 1, which may be due to the type of interaction between odor compounds and substrates (synergistic or antagonistic). In addition, when calculating OAV, it is assumed that the relationship between the concentration of each compound and OAV is linear. However, the concentration of odor compounds and the contribution of the substance to the overall aroma profile might not be completely linear, with certain configurations having a sigmoid function. Moreover, individual differences in odor intensity of aroma-active compounds must be taken into consideration [36], [37].

### 3.6 Aroma recombination assays

**Fig. 5:**
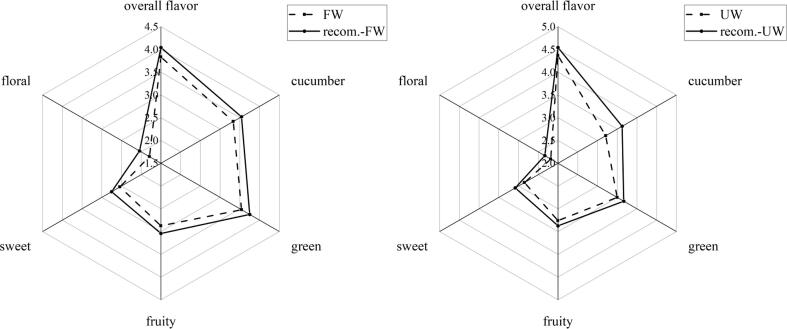
Aroma characteristics of watermelon juice and corresponding reconstituted samples.

To validate major aroma-active compounds identified herein in watermelon juice, aroma reorganization experiments were conducted. Reference odorant solutions of fourteen compounds identified in FW and 16 compounds identified in UW with OAV > 1 at concentrations similar to those revealed by quantitative analysis (Table 2) were mixed with a simulated watermelon juice model. The results of sensory analysis of aroma reconstituted samples and actual watermelon juice samples are shown in Fig. 5. The aroma profile of aroma reconstituted samples was similar to those of actual watermelon juice, although various sensory attributes in aroma reconstituted samples were starker. The sensory attributes (cucumber, green, fruity, sweet, and floral) conferred by the fourteen and sixteen major aroma-active compounds represented flavor characteristics of FW and UW, respectively.

### 3.7 Omission experiment assays

To explore the contribution of certain aroma-active compounds, the triangle test is applied by systematically omitting each compound of the 18 key aroma-active compounds, and the results are shown in Table 3. In the omitted experiment of the FW group, all panelists could detect the omission of hexanal, (*Z*)-6-nonenal, (*E*)-2-nonenal, and (*E*,*Z*)-2,6-nonadienal, suggesting that these compounds play a key role in determining the overall aroma of watermelon juice samples. However, when 3-methylbutanal, 2-hexenal, 6-methyl-5-hepten-2-one, hexanol, and geranyl acetone were removed, no significant effect on the reconstructed model was observed. Therefore, it could be further confirmed that hexanal, nonanal, (*Z*)-6-nonenal, (*E*)-2-nonenal, (*E*,*Z*)-2,6-nonadienal, nonanol, (*Z*)-6-nonenol, (*E, Z*)-3,6-nonadienol, and (*E*,*Z*)-2,6-nonadienol were the key aroma-active compounds in FW. In contrast, in the UW group, all panelists were able to detect the omission of limonene, nonanal, (*Z*)-6-nonenal, (*E*)-2-nonenal, and (*E*,*Z*)-2,6-nonadienal, suggesting the importance of these compounds in determining the overall aroma profile of UW samples. However, when 3-methylbutanal, 2-hexenal, hexanol, *β*-caryophyllene, (*E*,*Z*)-3,6-nonadienol and geranyl acetone were removed, no significant effect on the reconstructed model was observed. Therefore, these ten compounds were further confirmed as the key aroma-active compounds in UW.

**Table 3:** Omission experiment.

No.	Compounds a	n b		Significant c	
		FW	UW	FW	UW
1	(*Z*)-6-nonenal	12	12	***	***
2	(*E*)-2-nonenal	12	12	***	***
3	(*E*,*Z*)-2,6-nonadienal	12	12	***	***
4	limonene		10		***
5	nonanal	9	10	**	***
6	*α*-copaene		9		**
7	hexanal	10	9	***	**
8	nonanol	9	8	**	*
9	(*E*,*Z*)-2,6-nonadienol	9	9	**	**
10	(*Z*)-6-nonenol	8		*	
11	(*E*,*Z*)-3,6-nonadienol	8	7	*	–
12	*β*-ionone		8		*
13	6-methyl-5-hepten-2-one	7		–	
14	2-hexenal	4	7	–	–
15	3-methylbutanal	5	6	–	–
16	geranyl acetone	5	7	–	–
17	hexanol	3	5	–	–
18	*β*-caryophyllene		4		–

a Aroma-active compounds with OAV ≥ 1 in Table 2.

b Number of correct judgments from 12 assessors by the triangle test.

c Significance: “*”, significant (α ≤ 0.05); “**”, highly significant (α ≤ 0.01); “***”, very highly significant (α ≤ 0.001); “-”, non-significant.

## 4 Conclusion

In the present study, the aroma profile of FW and UW was characterized by molecular sensory analysis to understand the effect of ultrasonic treatment on the flavor of watermelon juice. Ultrasonic treatment could significantly enhance overall flavor of watermelon juice, especially related to floral, fruity, and sweet odor attributes. A total of 85 volatile compounds were identified in FW and UW by GC-O-MS. Furthermore, 12 key aroma-active compounds were identified by AEDA, OAV, aroma recombination, and omission experiments, among which 7 compounds contributed to the aroma profile of both FW and UW, namely hexanal, nonanal, (*Z*)-6-nonenal, (*E*)-2-nonenal, (*E*,*Z*)-2,6-nonadienal, nonanol and (*E*,*Z*)-2,6-nonadienol. Moreover, (*Z*)-6- nonenol and (*E*,*Z*)-3,6-nonadienol were the unique key aroma-active compounds in FW, whereas limonene, *α*-copaene, and *β*-ionone are the key aroma-active compounds specific to UW. Furthermore, the key aroma-active substances were the main substances accounting for the difference between the aroma profile of watermelon juice before and after ultrasonic treatment. These results provide additional information on watermelon juice flavor, and might serve as the basis for the rational utilization of watermelon resources, thus promoting the development of industrial watermelon processing.

## CRediT authorship contribution statement

**Fan Yang:** Investigation, Writing – original draft. **Chunhe Shi:** Writing – review & editing. **Lichang Yan:** Writing – review & editing. **Ying Xu:** Methodology, Formal analysis. **Yixin Dai:** Methodology, Formal analysis. **Shuang Bi:** Supervision. **Ye Liu:** Supervision, Project administration.

## Declaration of Competing Interest

The authors declare that they have no known competing financial interests or personal relationships that could have appeared to influence the work reported in this paper.
