# Supplementary material for: Low-frequency ultrasonic treatment: A potential strategy to improve the flavor of fresh watermelon juice
Source: Ultrason Sonochem. 2022 Nov 23;91:106238. doi: 10.1016/j.ultsonch.2022.106238 (PMC9703038; doi:10.1016/j.ultsonch.2022.106238)
Supplement: Supplementary data 1 [file mmc1.docx]

Supplement 1 Concentration gradient of aroma-active compounds

| Compounds | Concentration (μg/L) | | | | | | |
| --- | --- | --- | --- | --- | --- | --- | --- |
|  | 1 | 2 | 3 | 4 | 5 | 6 | 7 |
| 2-hexenal | 2.07 | 8.28 | 41.40 | 82.80 | 207.00 | 276.00 | 414.00 |
| hexanol | 2.17 | 8.17 | 40.85 | 81.70 | 204.25 | 272.33 | 408.50 |
| *β*-ionone | 2.33 | 9.32 | 46.60 | 93.20 | 233.00 | 310.67 | 466.00 |
| 3-methylbutanal | 8.00 | 20.00 | 40.00 | 80.00 | 200.00 | 266.67 | 400.00 |
| hexanal | 8.01 | 20.03 | 40.05 | 80.10 | 200.25 | 267.00 | 400.50 |
| limonene | 8.42 | 21.05 | 42.10 | 84.20 | 210.50 | 280.67 | 421.00 |
| 6-methyl-5-hepten-2-one | 8.55 | 21.38 | 42.75 | 85.50 | 213.75 | 285.00 | 427.50 |
| (*Z*)-6-nonenol | 8.45 | 21.13 | 42.25 | 84.50 | 211.25 | 281.67 | 422.50 |
| geranyl acetone | 8.73 | 21.83 | 43.65 | 87.30 | 174.60 | 291.00 | 436.50 |
| nonanal | 41.35 | 68.92 | 103.38 | 165.40 | 413.50 | 551.33 | 827.00 |
| (*Z*)-6-nonenal | 41.70 | 69.50 | 104.25 | 166.80 | 417.00 | 556.00 | 834.00 |
| *α*-copaene | 44.95 | 74.92 | 112.38 | 179.80 | 449.50 | 599.33 | 899.00 |
| (*E*)-2-nonenal | 41.70 | 69.50 | 104.25 | 166.80 | 417.00 | 556.00 | 834.00 |
| (*Z*)-3-nonenol | 42.25 | 70.42 | 105.63 | 169.00 | 422.50 | 563.33 | 845.00 |
| (*E*,*Z*)-2,6-nonadienal | 43.00 | 71.67 | 107.50 | 172.00 | 430.00 | 573.33 | 860.00 |
| *β*-caryophyllene | 45.05 | 75.083 | 112.6 | 180.20 | 450.50 | 600.67 | 901.00 |
| nonanol | 41.35 | 68.92 | 103.38 | 165.40 | 413.50 | 551.33 | 827.00 |
| (*E*,*Z*)-3,6-nonadienol | 43.00 | 71.67 | 107.50 | 172.00 | 430.00 | 573.33 | 860.00 |
| (*E*,*Z*)-2,6-nonadienol | 43.00 | 71.67 | 107.50 | 172.00 | 430.00 | 573.33 | 860.00 |
